# The Advantages and Disadvantages of Esmarch’s Bloodless Method

**Published:** 1875-03

**Authors:** 


### THE ADVANTAGES AND DISADVANTAGES OF ESMARCH'S BLOODLESS METHOD.

A paper on this new method was recently read before the County Medical Society, by Dr. Henry B. Sands. The object of the paper was to give a summary of the operations in which it was employed, in the city of New York and its vicinity, during the first year of its trial here, and in connection with the results obtained to devise an estimate of the value of the method. The record of instances in which it was employed comprised a list of 143 cases, tabulated so as to indicate the nature of the operation, and, in the fatal cases, the cause of death. Upon the whole, the experience appears to have been very favorable to the new method. In the only instances where evil results seemed to have been due to the application of the elastic bandage, they were more fairly attributable to the mode of its application than to the method itself. It was stated that certain advantages of the method were unquestionable. As to its bloodless character, Dr. Sands regards it as almost perfect, and says that there is only a loss of a few drops of blood during the operation, and the loss of blood from oozing, which occurs after the constricting band has been removed, is far less than the gain by this new method over the older method. After the completion of the operation, the patient has often a relatively greater supply of blood in his body than before the operation was commenced. An interesting point alluded to in this connection is the apparent impunity with which the vascular system suffers this sudden increase or tension. It is, however, suggested that, in case of thoracic or abdominal disease the sudden distension of the vessels with blood may possibly be attended with danger.

Besides the immediate advantage to the patient of the bloodless operation, the method becomes of vast service to the surgeon under circumstances where deep dissections are necessary, as for the removal of tumors or foreign bodies, or in searching for a deeply-seated wounded vessel. Under these circumstances a clear and unobstructed view of the tissues that come under the knife is very desirable. While the parts are stained and obscured by blood, important structures may easily be wounded or injured, which, with the aid of Esmarch's apparatus, might be safely avoided. This, however, does not wholly apply to the blood-vessels, their emptiness rendering them somewhat difficult to be recognized. As a precaution, therefore, it is advised that the operator "make good use of his anatomical knowledge, and study the appearance of the tissues before he divides them."

There is another use to which Esmarch's apparatus might be put, as observed by the writer, viz: in those cases where compound fractures are attended with free hemorrhage; and it is suggested that were ambulance surgeons and those in charge of the police stations supplied with the apparatus, it would frequently be the means of saving life.

The possible disadvantages of the bloodless method are considered under the heads of sloughing, secondary hemorrhage and paralysis. These mishaps all occurred in the cases collected, and in several instances were clearly due to the employment of Esmarch's method. Still in each of these instances there was reason to suppose that the method had not been properly applied. With regard to the applicability of the bandage, it was observed that it is desirable to abstain from its employment in certain cases, and above all to learn the minimum degree of pressure that will accomplish the desired result. The bandage should be soft and highly elastic, and the constriction of the limb should be made either by a piece of the same material, or, where this would be too wide, by a piece of soft rubber tubing. The solid cord should, he thinks, be abandoned, as likely to do mischief. The constriction should also not be applied for a longer time than absolutely necessary, the danger probably increasing with the length of time the pressure is continued. *N. Y. Medical Record.*

---

*Guaiacum in the Treatment of Acute Tonsilitis.*—Dr. R. J. Fritzinger (in the *Reporter* of Nov. 7th) recommends the following: ℞. Potass. chlor. ʒ j.; spts. nit. dulc., ℥ ss; tinct. guaiac, ℥ iss. Ft. sol. A teaspoonful every 3 hours, in sweetened water.

As the local action plays a prominent part in the cure, it always should be swallowed slowly. The use of astringent gargles should not be omitted.—*St. Louis Med. and Surg. Jour.*, Dec., 1874.

